# Remote Assessment of Cardiovascular Risk Factors and Cognition in Middle-Aged and Older Adults: Proof-of-Concept Study

**DOI:** 10.2196/30410

**Published:** 2022-02-02

**Authors:** Jennifer A Eastman, Allison R Kaup, Amber L Bahorik, Xochitl Butcher, Mouna Attarha, Gregory M Marcus, Mark J Pletcher, Jeffrey E Olgin, Deborah E Barnes, Kristine Yaffe

**Affiliations:** 1 San Francisco VA Medical Center San Francisco, CA United States; 2 Department of Psychiatry and Behavioral Sciences University of California San Francisco, CA United States; 3 The Neurology Center of Southern California Carlsbad, CA United States; 4 Department of Medicine University of California San Francisco, CA United States; 5 Posit Science Corporation San Francisco, CA United States; 6 Department of Epidemiology and Biostatistics University of California San Francisco, CA United States; 7 Department of Neurology University of California San Francisco, CA United States

**Keywords:** mHealth, internet, mobile health, digital health, eHealth, cardiovascular, risk factors, cognition, cognitive impairment, remote cognitive assessment, aging

## Abstract

**Background:**

Adults with cardiovascular disease risk factors (CVRFs) are also at increased risk of developing cognitive decline and dementia. However, it is often difficult to study the relationships between CVRFs and cognitive function because cognitive assessment typically requires time-consuming in-person neuropsychological evaluations that may not be feasible for real-world situations.

**Objective:**

We conducted a proof-of-concept study to determine if the association between CVRFs and cognitive function could be detected using web-based, self-administered cognitive tasks and CVRF assessment.

**Methods:**

We recruited 239 participants aged ≥50 years (mean age 62.7 years, SD 8.8; 42.7% [n=102] female, 88.7% [n=212] White) who were enrolled in the Health eHeart Study, a web-based platform focused on cardiac disease. The participants self-reported CVRFs (hypertension, high cholesterol, diabetes, and atrial fibrillation) using web-based health surveys between August 2016 and July 2018. After an average of 3 years of follow-up, we remotely evaluated episodic memory, working memory, and executive function via the web-based Posit Science platform, BrainHQ. Raw data were normalized and averaged into 3 domain scores. We used linear regression models to examine the association between CVRFs and cognitive function.

**Results:**

CVRF prevalence was 62.8% (n=150) for high cholesterol, 45.2% (n=108) for hypertension, 10.9% (n=26) for atrial fibrillation, and 7.5% (n=18) for diabetes. In multivariable models, atrial fibrillation was associated with worse working memory (β=-.51, 95% CI -0.91 to -0.11) and worse episodic memory (β=-.31, 95% CI -0.59 to -0.04); hypertension was associated with worse episodic memory (β=-.27, 95% CI -0.44 to -0.11). Diabetes and high cholesterol were not associated with cognitive performance.

**Conclusions:**

Self-administered web-based tools can be used to detect both CVRFs and cognitive health. We observed that atrial fibrillation and hypertension were associated with worse cognitive function even in those in their 60s and 70s. The potential of mobile assessments to detect risk factors for cognitive aging merits further investigation.

## Introduction

In-person neuropsychological evaluation through conventional paper-and-pencil tests administered one-to-one in a clinic or lab by a trained psychometrist represents the gold standard for testing cognitive function [1-3]. However, there are drawbacks associated with this method of evaluation including limited availability, high cost, lengthy time commitment for both study participants and personnel, and limited accessibility of even the most commonly used cognitive testing measures in community settings with hard-to-reach or at-risk populations. These factors alone are reason enough to seek out more efficient means of collecting cognitive data for large-scale research. In the face of a global pandemic, the vital importance of developing remote cognitive assessment tools for both research productivity and improved patient care has never been more apparent.

Remote cognitive assessment as a mobile health (mHealth) technology offers the potential of increased flexibility and portability, and improved efficiency in neuropsychological testing [4]. mHealth is the use of mobile technology including phones, tablets, and wearable devices to collect data and interface with patients for treatment or research [5]. It provides the unique opportunity to study health risk factors remotely through web-based platforms without in-person visits. Web-based cognitive testing can address the weaknesses of traditional neuropsychological evaluation by showing improved accuracy in administration and scoring, adaptability to the performance of the test taker, decreased cost of administration, shorter and more precise batteries, and an increased accessibility for diverse and rural populations [1,6,7].

Traditional, in-person studies of cardiovascular risk factors (CVRFs) such as diabetes mellitus, atrial fibrillation, hypertension, and high cholesterol have reported associations with higher risk for cognitive decline and dementia in older adulthood [8-13]. With this in mind, we developed the Health eBrain study. We sought to determine whether a self-administered mobile cognitive intervention tool could detect the associations between CVRFs and cognitive performance using completely remote measures. We hypothesized that mobile measures of cardiovascular health would be associated with mobile measures of cognition in adults aged ≥50, such that those with CVRFs would demonstrate significantly worse cognitive performance.

## Methods

### Recruitment

Health eHeart conducts enrollment, consent, and participation entirely through the internet. Participation is open to any individual aged 18 or older with an email address. Recruitment for Health eHeart is worldwide and accomplished through news stories, email, social media, and word of mouth [14]. Health eBrain was designed as a companion study with recruitment through Health eHeart.

### Statistical Analysis

To assess the association between CVRFs and cognitive performance, we sent email invitations to all Health eHeart Study participants enrolled from August 2016 to July 2018 who were aged 50 years or older, were English speaking, were not colorblind, and had internet access and sufficient computer proficiency to engage in online assessment. We used this time point as our goal was to assess cognition 2-3 years following enrollment. A total of 741 eligible individuals consented to participate in the study. Of these, 502 did not complete testing, and the final analytical cohort was 239 participants (mean age 62.74 years, SD 8.8; 42.7% [n=102] female, 88.7% [n=212] White), who completed the web-based testing between March 20, 2019, and May 31, 2019.

### CVRF Measurements

Hypertension, high cholesterol, and diabetes diagnoses were established through self-report of a physician’s diagnosis or by laboratory values obtained from self-report or mobile device monitoring and were defined by American Heart Association criteria for diabetes (fasting glucose >126 units), high blood pressure (BP >140/90 units), and high cholesterol (total cholesterol >240 units). Atrial fibrillation was identified through participant self-report of a physician’s diagnosis, a method previously validated in the Health eHeart cohort [15].

### Cognitive Assessment

Between March 2019 and June 2019, Health eBrain participants completed a series of cognitive tasks on the Posit Science BrainHQ platform [16], which was integrated with the Eureka Research Platform through an application programming interface. BrainHQ is a series of web-based, self-administered cognitive tasks engineered to run on a computer or as a mobile app on iOS devices. It provides game-like cognitive exercises, with the ability to assess the performance of individuals relative to their peers. Of the tasks available through BrainHQ, 6 were used to assess 3 cognitive domains: episodic memory, working memory, and executive function. One cognitive task (To and Fro Motor Speed) was used solely to adjust for motor speed processing.

While BrainHQ was originally designed around the concept of brain plasticity for the purpose of cognitive training and intervention, the study participants were not engaged in brain training. Each was given a single administration of the cognitive tasks linked to their unique email address. After completion, they received a summary of performance results from BrainHQ. Our data were audited to confirm the use of the primary testing session data and to prevent the analysis of repeat testing among participants who registered a second email address to improve their performance.

### Data Analysis

Descriptive statistics were used to characterize the sample. BrainHQ raw data were normalized (z-score) and averaged to derive cognitive domain scores. Table 1 presents the cognitive domains used in the study and details the BrainHQ tasks that comprised each domain. We then used unadjusted and adjusted linear regression models to examine associations between the CVRFs and each cognitive domain. Adjusted models included demographic characteristics (age, race, and sex). We also conducted a sensitivity analysis, which further corrected for a measure of motor speed processing. Tests of statistical significance were two-tailed with significance set to *P*<.05. The analyses were performed using R version 4.0.2 (R Foundation for Statistical Computing).

**Table 1:** Cognitive domains and associated tasks.

Cognitive domain or test		Description
**Episodic memory**		
	Face facts	Recall for names, faces, and facts
	Auditory paired associates	Auditory recognition task
	Pathfinder	Visuospatial learning and memory
Working memory	Target tracker	Multiple-object target tracking
**Executive function**		
	Word conflict	Stroop task
	Rule switcher	Color or shape rule-switching for set shifting
Motor speed adjustment	To and Fro test	Test of motor speed

## Results

At the time of cognitive testing, the 239 participants had a mean age of 62.74 years (SD 8.8). Moreover, 42.7% (n=102) were female, and 88.7% (n=212) had non-Hispanic White racial backgrounds. CVRF prevalence was 62.8% (n=150) for high cholesterol, 45.2% (n=108) for hypertension, 10.9% (n=26) for atrial fibrillation, and 7.5% (n=18) for diabetes (Table 2).

**Table 2:** Participant characteristics.

Characteristics		Values
Age (years), mean (SD)		62.7 (8.8)
	<60	101 (42.3)
	60-70	87 (36.4)
	>70	51 (21.3)
Gender, n (%)	Female	102 (42.7)
**Race, n (%)**		
	White	212 (88.7)
	Black	8 (3.3)
	Asian	10 (4.2)
	Other	7 (2.9)
	Not disclosed	2 (0.8)
**Cardiovascular risk factors, n (%)**		
	High cholesterol	150 (62.8)
	Atrial fibrillation	26 (10.9)
	Diabetes	18 (7.5)
	Hypertension	108 (45.2)
**Cognitive assessment, n (%), range**		
	Episodic memory	17.4 (6.3), 5.2 to 32.4
	Working memory	4.4 (0.7), 2.8 to 5.8
	Executive function	211.7 (111.2), -56.2 to 515

In unadjusted models, the participants with atrial fibrillation performed more poorly than those without atrial fibrillation on measures of episodic memory (β=-.31, 95% CI -0.59 to -0.04) and working memory (β=-.51, 95% CI -0.91 to -0.11), and a trend association was observed for executive function (β=-.33, 95% CI -0.74 to -0.07). The participants with hypertension performed more poorly than those without hypertension on measures of episodic memory (β=-.27, 95% CI -0.44 to -0.11), with a trend association for working memory (β=-.22, 95% CI -0.47 to 0.03) and no association for executive function (β=.03, 95% CI -0.22 to 0.28). There was no significant association between high cholesterol or diabetes and cognitive test performance. After adjustments for sex, age, and race, the findings remained unchanged from those of the unadjusted model (Table 3).

**Table 3:** Associations between cardiovascular risk factors and cognitive performance.

Cognitive performance according to CVRF^a,b^		Unadjusted β (95% CI)	*P* value	Demographics-adjusted^c^ β (95% CI)	*P* value
**Atrial fibrillation**					
	Episodic memory	-.31 (-0.59 to -0.04)	.02	-.28 (-0.54 to -0.02)	.03
	Working memory	-.51 (-0.91 to -0.11)	.01	-.49 (-0.89 to -0.09)	.01
	Executive function	-.33 (-0.74 to -0.07)	.10	-.31 (-0.71 to 0.09)	.12
**Hypertension**					
	Episodic memory	-.27 (-0.44 to -0.11)	<.001	-.20 (-0.36 to -0.04)	.01
	Working memory	-.22 (-0.47 to 0.03)	.09	-.15 (-0.40 to 0.10)	.24
	Executive function	.03 (-0.22 to 0.28)	.80	.10 (-0.14 to 0.36)	.40
**High cholesterol**					
	Episodic memory	-.01 (-0.18 to 0.16)	.91	.02 (-0.14 to 0.19)	.78
	Working memory	-.08 (-0.34 to 0.17)	.52	-.07 (-0.33 to 0.18)	.58
	Executive function	-.13 (-0.39 to 0.12)	.31	-.10 (-0.36 to 0.16)	.44
**Diabetes**					
	Episodic memory	-.23 (-0.55 to 0.08)	.15	-.14 (-0.45 to 0.16)	.27
	Working memory	.07 (-0.40 to 0.56)	.75	.18 (-0.29 to 0.66)	.45
	Executive function	-.29 (-0.77 to 0.18)	.22	-.19 (-0.67 to 0.28)	.43

^a^CVRF: cardiovascular risk factors.

^b^Standardized difference in cognitive test (95% CI)

^c^Adjusted for age, race, and sex.

In a sensitivity analysis, we further adjusted for a measure of motor speed processing derived from the To and Fro cognitive task and found almost identical results.

## Discussion

The results of this proof-of-concept study suggest that mHealth assessment tools can be used to effectively detect the association between CVRFs and cognitive function. We were successful at recruiting, consenting, and measuring the participants’ cognitive function all by remote assessment. Furthermore, we found that those with atrial fibrillation and hypertension had worse cognitive performance in the area of memory.

Our investigation contributes to the field by examining the mHealth data and cognitive performance of middle-aged to older adults entirely through remote technology. While numerous studies have observed the utility of mHealth tool across a spectrum of neurological conditions (eg, dementia, stroke, and multiple sclerosis) [3,4,6,7,17-19], our study is unique in that we demonstrated that this technology can be used to identify subtle cognitive deficits in otherwise healthy middle-aged and older adults, without in-person patient contact. Additionally, our results contradict the perception that older adults do not or cannot participate in technology-driven mHealth assessment.

Our findings are consistent with in-person neuropsychological testing results that have demonstrated that individuals with CVRFs are at increased risk for cognitive decline and dementia as they age [9,13,20,21]. Specifically, atrial fibrillation absent stroke has been associated with cognitive decline and dementia and may accelerate cognitive decline through mechanisms of hypoperfusion, systemic inflammation, and cerebral small vessel diseases [22,23], while hypertension has been associated with increased risk for cognitive decline and microstructural white matter alterations [24,25]. The results for high cholesterol have been mixed [26-30]; however, many studies have reported an association with diabetes and worse cognition. Our lack of finding an association with diabetes may have been due to limited power as only 7% (n=18) had diabetes.

The high CVRF prevalence in the US population is concerning. The Third National Health and Nutrition Examination Survey (NHANES III) estimates that 60% of men and 50% of women have 1 to 2 CVRFs, and this increases with age [31,32]. Given these striking numbers, mHealth studies such as Health eHeart and remote cognitive assessment tools may represent a viable strategy for closely tracking and monitoring the cognitive effects of CVRFs in at-risk patients, with the hope of improving outcomes.

The Health eBrain proof-of-concept study utilized well-established platforms to remotely evaluate the relationship between CVRF exposure and cognition. Despite the study’s strengths, several limitations need to be considered. Our sample size was relatively small, particularly regarding participants with diabetes, and this may have reduced our ability to detect subtle cognitive changes. Second, our cohort was not very diverse as compared to the US population. This is in part a reflection of the Health eHeart Study cohort, which is less likely to be from Black, Hispanic, or Asian (versus White or non-Hispanic) racial backgrounds, relative to all adults in the United States [32]. This lack of diversity in Health eHeart may be due to a recruitment strategy that lacks the specific targeting of minority populations. It also may be due to the “digital divide” and associated issues with access to technology related to socioeconomic and cultural factors. While our results indicate that middle-aged and older adults can actively engage in the use of cell phone and computer technologies to participate in remote research and health tracking, our participants may represent an overall higher-functioning group relative to their peers. Therefore, our results may not be generalizable to the broader population. Finally, while remote, self-guided cognitive assessments have a great potential to provide large-scale cognitive data, they are not without their weaknesses. Control of the testing environment is an important component to standardizing and interpreting results to prevent distractions that can artificially lower the testing scores. Cognitive performance in remote testing is vulnerable to extraneous circumstances, level of task engagement, display of emotion and frustration, or the tendency to give up easily, and this study cannot account for that variability [3].

The results suggest that mHealth assessments can be used to detect the association between CVRFs and cognition function. mHealth tools demonstrated specific sensitivity for detecting memory deficits associated with atrial fibrillation and hypertension in middle-aged and older adults. Future research would benefit from a larger study of this remote assessment platform with longitudinal monitoring of CVRFs and cognitive performance. The ability to remotely track cognitive health in individuals with modifiable CVRFs could represent a unique opportunity to target high-risk individuals for early education, frequent monitoring, and interventions with the hope of preventing accelerated cognitive decline with aging.

## Abbreviations

CVRF: cardiovascular risk factor
mHealth: mobile health
NHANES III: Third National Health and Nutrition Examination Survey
